# Report of case series: Correlation between pathological and radiological evaluation and clinical course of three cases of metastatic renal cell carcinoma with cytoreductive nephrectomy after combined immuno‐oncology therapy

**DOI:** 10.1002/iju5.12752

**Published:** 2024-06-16

**Authors:** Yuichiro Atagi, Kouki Tada, Reina Kouno, Ryoei Minato, Katsuyoshi Hashine

**Affiliations:** ^1^ Department of Urology NHO Shikoku Cancer Center Matsuyama Ehime Japan

**Keywords:** cytoreductive surgery, immunotherapy, nephrectomy, renal cell carcinoma, sarcomatoid

## Abstract

**Introduction:**

The pathologic evaluation and clinical course of cytoreductive nephrectomy after combined immuno‐oncology therapy were reviewed to understand the benefits of cytoreductive nephrectomy.

**Case presentation:**

Three patients with clear cell carcinoma underwent tumor biopsy before combined immuno‐oncology therapy. Case 1 was found to have a sarcomatoid component upon nephrectomy and continued with combined immuno‐oncology therapy. Case 2 discontinued combined immuno‐oncology therapy due to adverse events but maintained tumor shrinkage. The patient was found to have viable cells in most nephrectomy specimens but has had no recurrence after combined immuno‐oncology therapy was discontinued. In case 3, the residual tumor was deemed resectable with combined immuno‐oncology therapy, and nephrectomy and metastasectomy were performed. No viable cells were observed in either specimen, and the patient has had no recurrence.

**Conclusion:**

Cytoreductive nephrectomy after combined immuno‐oncology therapy may be useful to allow pathologic evaluation of treatment and provide an indicator for subsequent treatment.

**Abbreviations & Acronyms**
Axi: axitinib
CN: cytoreductive nephrectomy
CT: computed tomography
IMDC: International mRCC Database Consortium
IO: immuno‐oncology
Ipi: ipilimumab
irAE: immune‐related adverse event
irPR: immune‐related pathologic response
MPR: major pathologic response
mRCC: metastatic renal cell carcinoma
Nivo: nivolumab
ORR: overall response rate
OS: overall survival
Pem: pembrolizumab
PFS: progression‐free survival
RCC: renal cell carcinoma
TKI: tyrosine kinase inhibitor

**Keynote message**

Deferred CN with combined IO therapy may provide guidance for the next treatment strategy.

## Introduction

The SURTIME trial showed that deferred CN did not improve the progression‐free period in mRCC.^1^ However, a report on CN combined with the IO therapeutic sunitinib showed that deferred CN prolonged OS more than immediate CN.^2^ Therefore, we hypothesized that some patients may benefit from deferred CN when combined with IO therapy in mRCC. In this study, we retrospectively observed metastatic RCC cases treated with combined IO therapy to determine the relationship between the efficacy of combined IO therapy and subsequent courses of treatment.

## Case presentation

For each case, age, sex, TNM stage, IMDC risk classification, metastatic sites, combined IO therapy, and treatment efficacy are summarized in Table 1. In all cases, tumor biopsies were performed prior to combined IO therapy to diagnose clear cell RCC.

**Table 1:** Patient characteristics. The length of treatment is defined as the period up to the preoperative TKI withdrawal for cases 1 and 3, while case 2 was withdrawn for irAEs, and the length of treatment represents the treatment period up to that point. PFS was defined as the period without tumor progression after CN

	Age	Sex	Clinical stage (TNM)	IMDC risk classification	Risk factors*	Metastatic sites	Combined IO therapy^†^	Length of treatment (weeks)	Treatment Effect (RECIST1.1)	Nephrectomy	Treatment after nephrectomy	PFS (months)
Case1	51	Male	T3aN0M1	Poor	Diagnosis, Hb, Ca	Lungs	Pem + Axi	6	19% reduction (SD)	Open	Pem + Axi → metastasectomy → Cabozantinib (on‐going)	12
Case2	61	Female	T3aN0M1	Poor	Diagnosis, Hb, Plt, Ca	Lungs, Pleura, External iliac lymph nodes	Ipi + Nivo	9	39% reduction (PR)	Laparoscopic	None	40
Case3	51	Male	T3aN0M1	Intermediate	Diagnosis, Ca	Clavicle, Scapula, Sacrum	Pem + Len	13	12% reduction (SD)	Laparoscopic	Pem + Len (ongoing)/metastasectomy	10

* Diagnosis: time from diagnosis to treatment, Hb: hemoglobin, Ca: corrected serum calcium, Plt: platelets.

^†^ Pem + Axi: pembrolizumab plus axitinib, Ipi + Nivo: ipilimumab plus nivolumab, Pem + Len: pembrolizumab plus lenvatinib.

### Case 1

**Fig. 1:**
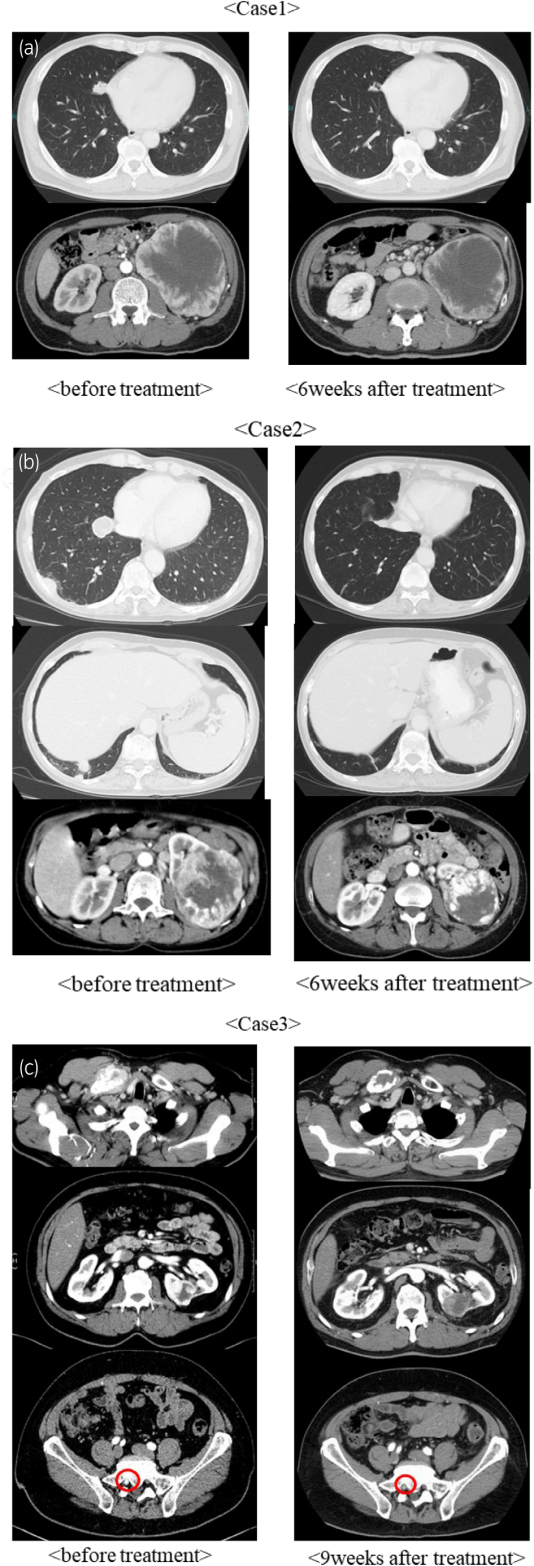
(a) Contrast CT of case 1 before and after the start of treatment. The primary tumor in the left kidney and metastases in the right lung are shown. The primary tumor did not shrink significantly, but the lung metastases were reduced. (b) CECT of case 2 before and after the start of treatment. The primary tumor in the left kidney and metastases in the right lung and pleura are shown. The lungs had multiple metastases. The treatment resulted in shrinkage of the tumor, and most of the lung metastases disappeared. (C) CECT before and after the start of treatment in case 3, showing the primary tumor in the left kidney and metastases in the left clavicle, left scapula, and sacrum. The sacral metastases are located near the neural foramen, circled in red. Posttreatment, the scapular and sacral metastases had disappeared. The kidney and clavicle metastases remained the same size, but the internal contrast effect was diminished.

**Fig. 2:**
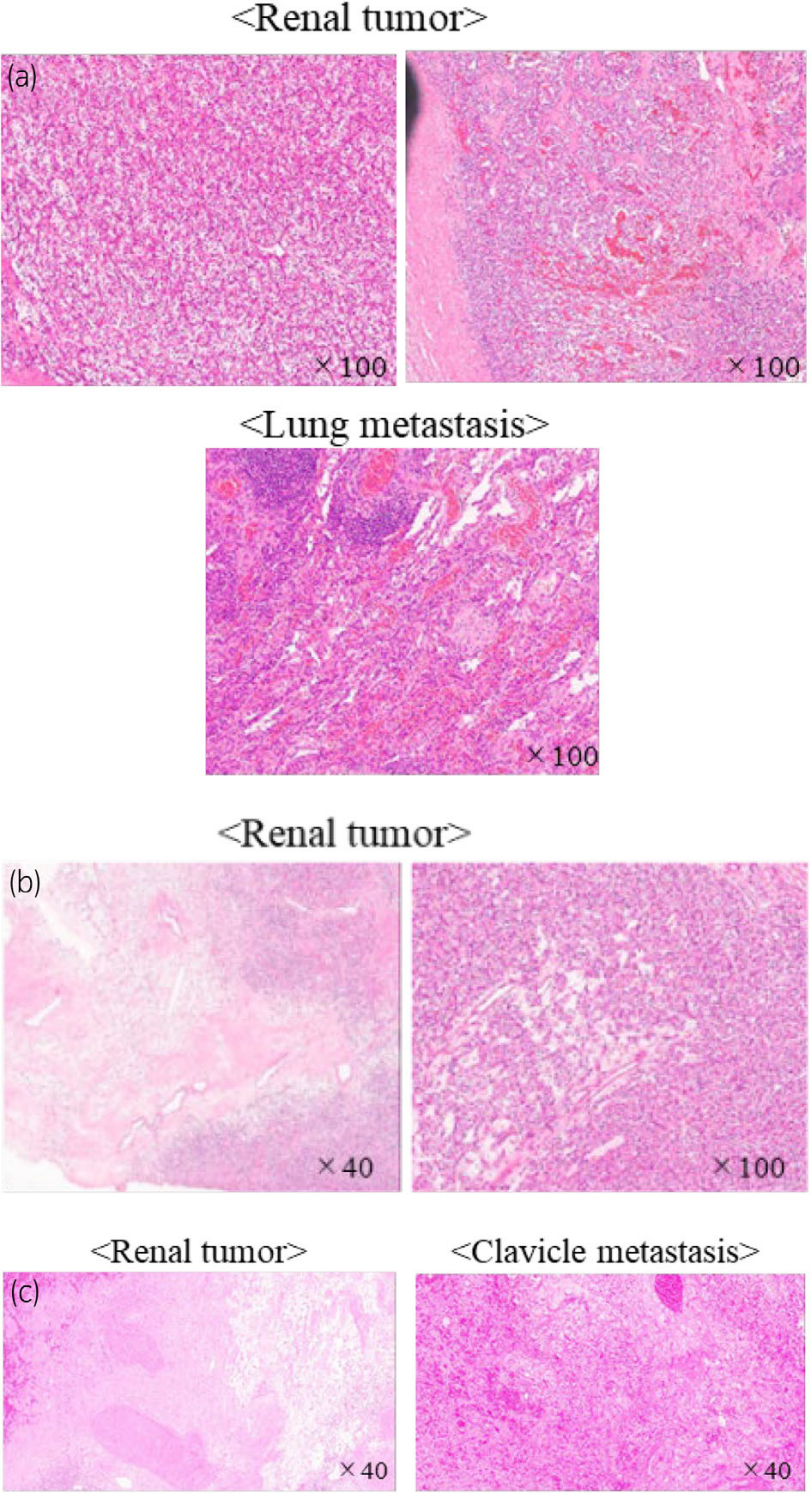
(a) Pathological specimens of the primary renal and pulmonary metastases in case 1 show the left side of the renal tumor, which is mostly a clear cell RCC, and the right side with sarcomatoid changes. Lung metastasectomy was performed using Pem + Axi 10 months after nephrectomy, and no residual clear cell RCC component remained, only sarcomatoid features. (b) Pathology specimen of the primary renal lesion in case 2. The left side shows inflammatory cell infiltration and some fibrosis, while the right side shows mostly residual tumor. (c) Pathology specimens of the primary renal and clavicle metastases in case 3. Both showed inflammatory cell infiltration with fibrous and scar tissue on the left side but no residual tumor.

The patient presented with cough, weight loss, and anemia, and was referred to us for a left renal tumor and multiple lung metastases. He was symptomatic, and pembrolizumab plus axitinib (Pem + Axi) was initiated as the first‐line treatment. Six weeks after starting Pem + Axi, CECT showed that lung metastases had decreased, but renal tumors had not changed significantly (Fig. 1a). We determined that the residual tumor was resectable, and 2 months after Pem + Axi initiation, the patient underwent an open left nephrectomy after a 1‐week withdrawal of axitinib. Pathology showed mostly clear cell RCC, but sarcomatoid lesions were also observed (Fig. 2a). Four weeks after nephrectomy, a CT scan showed increased lung metastases. We resumed Pem + Axi, and the lung metastases diminished but then increased 10 months after the resumption of treatment. We proceeded with surgical resection and performed a thoracoscopic lung metastasectomy 12 months after the resumption of treatment. The only remaining lung lesion after resection was a sarcomatoid lesion of renal cancer (Fig. 2a). A CT scan 3 weeks postoperatively showed residual tumor growth, and we initiated cabozantinib at 4 weeks postoperatively. The metastases diminished with cabozantinib, which is still being continued.

### Case 2

The patient was referred for cough, a left renal tumor, and multiple lung masses, and she was initially treated with ipilimumab plus nivolumab (Ipi + Nivo) because of her poor risk profile, minor symptoms, and young age. Six weeks after Ipi + Nivo treatment, CECT showed that most of the lung metastases had resolved, and the remaining tumors had decreased. Figure 1b (right side) shows all remaining lung metastases and that the renal tumor had decreased substantially. After three courses of Ipi + Nivo, the patient developed interstitial pneumonia grade 3 as an irAE. Following the initiation of prednisolone 50 mg, interstitial pneumonia was improved. Ipi + Nivo was stopped for over 3 months, and tumor shrinkage was maintained (Fig. 1b). Laparoscopic left nephrectomy was performed 6 months after Ipi + Nivo initiation. The pathological results showed that viable cells remained (Fig. 2b). We are following up to consider resuming treatment if the remaining metastases grow or new lesions appear. However, 3 years after CN, the tumor has not grown, and the patient is alive.

### Case 3

The patient presented with a mass on his right clavicle, a left renal mass, and multiple metastatic bone tumors. Although asymptomatic, pembrolizumab plus lenvatinib (Pem + Len) was initiated, which was highly effective in reducing tumor size. Nine weeks after initiation of Pem + Len treatment, CECT showed that the scapular and sacral tumors had disappeared. There was no reduction in renal or clavicular metastases, but there was a decrease in the contrast effect (Fig. 1c). Because the residual tumors appeared resectable, we performed CN and clavicle tumor metastasectomy 3 and 6 months after starting Pem + Len, with withdrawal of lenvatinib 1 week before surgery. The pathological results showed that there was no residual tumor (Fig. 2c). Four weeks after each surgery, we resumed Pem + Len, and 6 months after the resumption, there has been no recurrence and the patient remains alive.

## Discussion

In a recent report, long‐term survival was predicted if the immune‐related pathologic response was considered a MPR (residual viable tumor <10%).^3^ Here, only one patient had an MPR. However, case 2, in which MPR was not obtained, is nevertheless considered to have a “durable response” because the patient has been treatment‐free for 3 years. The renal tumors were large and had metastasized, which may have resulted in the reduction of the metastases but not the primary renal tumor. Furthermore, it is also known that the extracellular environment of each metastatic tissue is different and that the expression of various proteins is regulated during metastasis.^4^ Therefore, it is possible that the tumor environment, including the immune component, may differ between metastatic tumors and primary tumors, which may account for the observed therapeutic effect.

The MASS criteria, which are based on marked central necrosis (>50%), marked decreased attenuation (≥40 HU), or decreased size of more than 20%, were used and correlated with PFS.^5^ Previous reports of nephrectomy after combined IO therapy that can be evaluated by the MASS criteria are summarized in Table 2.^6^, ^7^, ^8^ No viable cells were found in case 3, in which MASS criteria were met.

**Table 2:** Three cases could be evaluated with MASS criteria based on images published in previous reports. MASS criteria also correlate with prognosis in CN after combined IO therapy. PFS was defined as the period without tumor progression after CN

Case	Authors	Reported year	Age	Sex	IMDC risk	Pathology	Combined IO therapy^†^	MASS criteria	Pathologic response	PFS (months)
1	Nakagawa *et al*.	2022	59	Male	Intermediate risk	Clear cell	Ipi + Nivo	Favorable response	MPR	6
2	Okada *et al*.	2020	47	Male	Intermediate risk	Clear cell	Ipi + Nivo	Favorable response	Pathological CR	6
3	Peak *et al*.	2020	60	Male	Intermediate risk	Clear cell	Ipi + Nivo	Favorable response	Pathological CR	8
4	Our case	2023	51	Male	Poor risk	Clear cell with sarcomatoid components	Pem + Axi	Intermediate response	Remaining viable tumors of more than 10%	1
5	Our case	2023	61	Female	Poor risk	Clear cell	Ipi + Nivo	Favorable response	Remaining viable tumors of more than 10%	36
6	Our case	2023	51	Male	Intermediate risk	Clear cell	Pem + Len	Favorable response	Pathological CR	10

^†^ Pem + Axi: pembrolizumab plus axitinib, Ipi + Nivo: ipilimumab plus nivolumab, Pem + Len: pembrolizumab plus lenvatinib.

While the initial tumor biopsy of the patient with an intermediate response (case 1) showed only clear cell carcinoma, performing CN revealed that the patient also had a sarcomatoid component with a pathologically poor prognosis. Metastasectomy was performed when only the sarcomatoid component remained. This suggests that CN may be useful in assessing treatment response and histomorphology for mRCC patients with an intermediate response by MASS criteria and in determining the next course of therapy.

Although diagnosed as clear cell carcinoma, 18.5% of cases are associated with sarcomatoid changes in their nephrectomy specimens.^9^ Sarcomatoid RCC is associated with a higher expression of PD‐L1 and a better response to IO therapy.^10^ First‐line therapy for sarcomatoid RCC with combined IO therapies resulted in an ORR of 55%–61% and PFS of approximately 11.1 months.^11^, ^12^, ^13^, ^14^ These interventions are inferior to the therapeutic effect achieved for clear cell RCC. This suggests that surgical resection should be aggressively performed, if possible, to determine the presence of a sarcomatoid component in the residual tumor.

Based on the above, deferred CN is beneficial in that it allows pathological evaluation, but the possibility of overtreatment should be avoided. The use of MASS criteria may provide a more accurate assessment of residual tumor on imaging. We hope that accumulating more cases in the future will enable us to make more accurate evaluations.

## Conclusion

Deferred CN may provide guidance for the next treatment strategy, including the evaluation of treatment after combined IO therapy.

## Author contributions

Yuichiro Atagi: Conceptualization; writing – original draft. Kouki Tada: Data curation. Reina Kouno: Data curation. Ryoei Minato: Data curation. Katsuyoshi Hashine: Writing – review and editing.

## Conflict of interest

No conflicts of interest.

## Approval of the research protocol by an Institutional Reviewer Board

The protocol for this research project has been approved by a suitably constituted ethics committee of the institution, and it conforms to the provisions of the Declaration of Helsinki (Ethics Review Committee of Shikoku Cancer Center, approval No. 2023‐518). Informed consent was obtained from all the subjects.

## Informed consent

Informed consent was obtained from the patients and their families.

## Registry and the Registration No. of the study/trial

Not Applicable.

## Data Availability

Data from this case report is available from the corresponding author upon reasonable request.
